# Optimized MaxEnt analysis revealing the change of potential distribution area of *Lygodium japonicum* in China driven by global warming

**DOI:** 10.3389/fpls.2025.1601956

**Published:** 2025-06-19

**Authors:** Yuxin Shan, Hongjian Shen, Ling Huang, Hamizah Shahirah Hamezah, Rongchun Han, Xia Ren, Chunhong Zhang, Xiaohui Tong

**Affiliations:** ^1^ School of Pharmacy, Anhui University of Chinese Medicine, Hefei, China; ^2^ Institute of Systems Biology, Universiti Kebangsaan Malaysia, Bangi, Malaysia; ^3^ Joint Research Center for Chinese Herbal Medicine of Anhui of IHM, Bozhou Vocational and Technical College, Bozhou, China; ^4^ Affiliated Taihe Hospital of Chinese Medicine, Anhui University of Chinese Medicine, Taihe, China; ^5^ School of Life Sciences, Anhui University of Chinese Medicine, Hefei, China; ^6^ Department of Research and Development, Functional Activity and Resource Utilization on Edible and Medicinal Fungi Joint Laboratory of Anhui Province, Lu’an, China

**Keywords:** Lygodium japonicum, MaxEnt, global warming, environment variables, potential distribution

## Abstract

*Lygodium japonicum* is a valuable medicinal plant with increasing demand in China, yet large-scale cultivation remains limited, relying heavily on wild populations. As climate change accelerates, its potential distribution is expected to shift, affecting suitable growth areas. Despite its medicinal importance, research on its adaptability and future habitat changes is limited. This study used an optimized MaxEnt ecological niche model and Geographic Information System (GIS) to predict the potential suitable habitats of *L. japonicum* under current and future climate conditions (2041-2060 and 2061-2080) across three greenhouse gas emission scenarios (SSP126, SSP245, SSP585). Results show that under current climatic conditions, the potential habitat of *L. japonicum* spans approximately 216.31 × 10^4^ km², with high suitability areas concentrated in southern and eastern China. In future climate scenarios, While the total suitable habitat area remains stable, the area of high suitability is significantly reduced. Specifically, under the SSP126 scenario, high suitability areas are projected to decrease by 44.1% during 2041-2060. The centroid of high suitability areas is expected to shift northward, though a localized southward shift is observed under the SSP126 scenario. Key environmental factors influencing the species’ distribution include temperature seasonality (bio4), May precipitation (prec5), and mean diurnal temperature range (bio2). These findings highlight the potential impacts of climate change on *L. japonicum*’s distribution and are crucial for the conservation and sustainable utilization of the species in China, particularly under changing climatic conditions.

## 1 Introduction

Global climate change is a critical issue, and the loss of biodiversity caused by both human activities and climate change poses significant challenges to ecosystems (Mantyka-Pringle et al., 2012). Plant growth and development are strongly influenced by climate factors such as temperature, precipitation, and humidity, which ultimately determine the geographical distribution of plant species (Bertrand et al., 2011). Global warming is driving an upward shift in the altitudinal distribution of many plant species. For example, in Southern California, warming has caused plant species to migrate upward by approximately 65 m (Kelly and Goulden, 2008). However, some mountain species in California have shifted to lower altitudes, possibly due to changes in the region’s water balance (Crimmins et al., 2011). Climate change also affects the spatial distribution of species across latitudes and longitudes. In North America, tree species are migrating northward as a result of warming and seasonal changes in precipitation (Harsch and HilleRisLambers, 2016). In Europe, while most species are migrating toward the poles, some have shifted eastward or westward due to factors such as nitrogen deposition (Sanczuk et al., 2024). Understanding these distribution shifts is essential for predicting future biodiversity trends and developing adaptive conservation strategies (Thorn et al., 2009).

Species Distribution Models (SDMs) are essential tools in ecology and conservation biology for predicting the potential distribution of species under specific environmental conditions. The Maxent model, based on the principle of maximum entropy, has gained significant attention due to its high predictive accuracy and flexibility (Elith et al., 2011). Maxent has been shown to outperform other models, such as BIOCLIM (bioclimatic prediction system) (Busby, 1991), DOMAIN (environmental domain analysis model) (Carpenter et al., 1993), and GARP (genetic algorithm for rule-set prediction) (Stockman et al., 2006), particularly when dealing with small sample sizes. Moreover, Maxent effectively addresses sampling bias (Fourcade et al., 2014), further enhancing its predictive performance (Terribile et al., 2010). These advantages make Maxent a powerful tool for research in ecology and conservation biology.

*Lygodium japonicum* (Thunb.) Sw., a perennial herbaceous vine of the Lygodiaceae family, is widely distributed across China, particularly in the southern and southwestern regions (Chen et al., 2010). This plant reproduces via spores, which are highly sensitive to environmental factors such as temperature during germination and growth (Kwon et al., 2016). Its dried spores are commonly used in traditional Chinese medicine for their heat-clearing, detoxifying, and diuretic properties, often treating urinary tract infections, urolithiasis, and jaundice. Additionally, *L. japonicum* contains bioactive compounds, including flavonoids, phenolic acids, and triterpenoids, which contribute to its antioxidant, antibacterial, and anti-inflammatory effects (Yang et al., 2021). With the growing demand for *L. japonicum* in the Chinese market, ensuring the sustainable use of this valuable resource has become critical. However, despite its recognized medicinal value, large-scale cultivation remains limited, with the plant mainly relying on wild populations and manual harvesting (Tong et al., 2023). As climate change accelerates, plant distributions are undergoing significant shifts (Hu et al., 2015; Shan et al., 2024; Wang et al., 2024), which could substantially affect the suitable growing conditions for *L. japonicum*, altering its potential distribution. Despite this, research on its ecological adaptability and future habitat shifts remains limited. Therefore, studying its adaptability and distribution dynamics under different climate conditions is essential for both conserving this wild medicinal resource and supporting the sustainable development of Chinese herbal medicine.

This study utilizes the Maxent model and ArcGIS technology to assess potential distribution changes of *L. japonicum* in China under current and future climate scenarios, focusing on its ecological adaptations. The aim is to provide scientific evidence for the sustainable use and conservation of *L. japonicum*, offering insights into effective adaptation strategies and habitat protection in the face of climate change. Ultimately, the study seeks to contribute to the sustainable utilization and conservation of *L. japonicum*, addressing the growing market demand.

## 2 Materials and methods

### 2.1 Distribution records and data processing of *L. japonicum*

**Figure 1:**
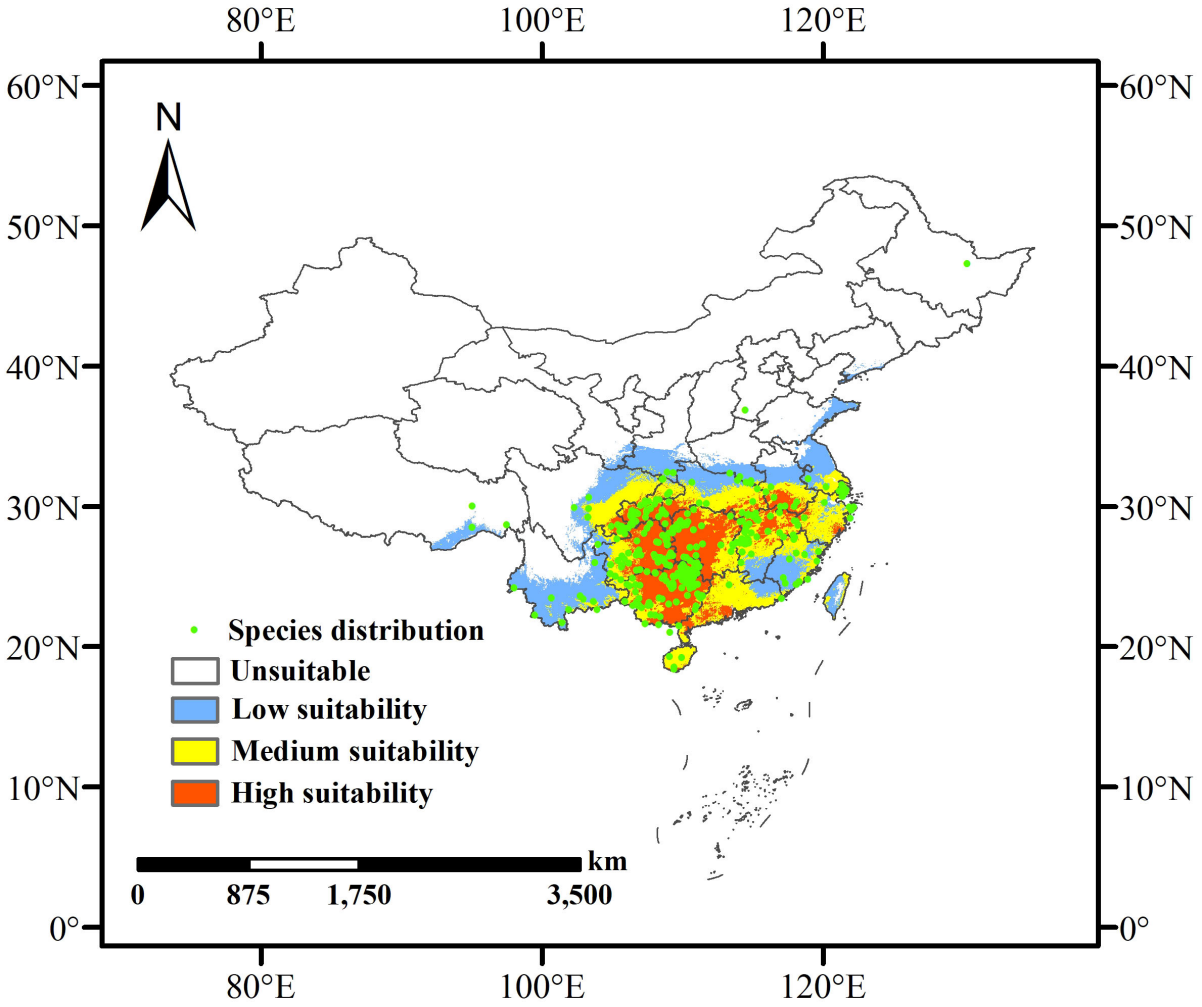
Predicted current habitat distribution of *L. japonicum* in China based on existing occurrence records.

Data from the Global Biodiversity Information Facility (GBIF, http://www.gbif.org) and the China Virtual Herbarium (CVH, http://www.cvh) were used to identify and record the locations of *L. japonicum* samples in China. After excluding duplicate coordinates, records lacking latitude and longitude information, and specimens dated before the 21st century, ENMTools.pl (https://github.com/danlwarren/ENMTools) was used to filter the occurrence points, retaining only one record per 2.5 arc-minute grid cell (Li et al., 2023). This process resulted in 337 records of *L. japonicum* in China, which were saved in CSV format and used to create detailed distribution maps in ArcGIS 10.8 (**Figure 1**).

### 2.2 Environmental variable data sources and preprocessing

To assess the factors influencing the distribution of *L. japonicum*, a total of 49 environmental variables were selected, including 19 bioclimatic variables, 24 monthly factors (precipitation and average temperature from January to December), 3 topographic factors, and 3 soil factors. Climate data, including monthly precipitation and average temperature (from January to December), as well as the topographic factors, were sourced from the WorldClim database (https://worldclim.org). The bioclimatic variables include both current and future climate data (2041-2060, 2061-2080). The 19 commonly used bioclimatic variables are labeled bio1 to bio19. For future projections, climate data from the BCC-CSM2-MR model of the Sixth Coupled Model Intercomparison Project (CMIP6) were used (O’Neill et al., 2017). Three Shared Socioeconomic Pathways (SSPs) were selected for analysis and simulation: SSP126 (low greenhouse gas emissions), SSP245 (medium greenhouse gas emissions), and SSP585 (high greenhouse gas emissions). The three soil factors were downloaded from the Harmonized World Soil Database (HWSD, https://www.fao.org/soils-portal/data-hub/soil-maps-and-databases/harmonized-world-soil-database-v12/en). All variables have a spatial resolution of 2.5 arc-min and were collected nationwide using ArcGIS.

**Figure 2:**
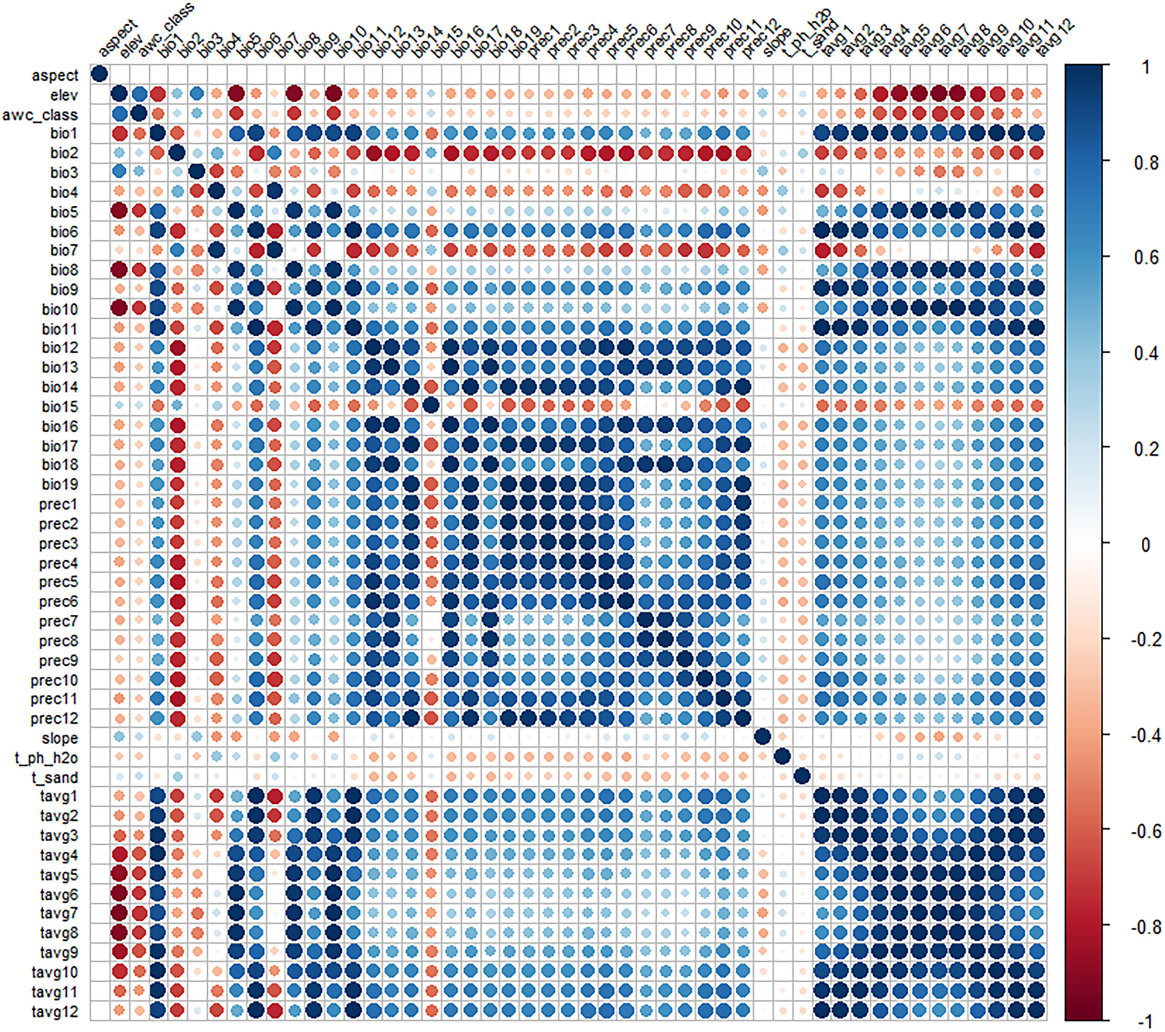
Correlation analysis of environmental factors.

To reduce the high correlation between environmental variables, which could lead to overfitting (Sillero, 2011), we first evaluated the contribution of each environmental factor to the model predictions using the jackknife test (a module in MaxEnt). Environmental factors with zero contribution were excluded. For the remaining factors, we analyzed their correlations using ENMTools.pl (**Figure 2**). A Pearson correlation coefficient threshold of 0.75 was adopted (Santana et al., 2019), and environmental factors with correlations greater than or equal to 0.75 were considered highly correlated. These highly correlated factors were then combined based on their contribution rates from the jackknife test, prioritizing those with higher contributions. Following this screening process, 12 environmental factors (bio2, bio4, bio7, bio14, slope, elev, prec3, prec4, prec5, prec6, prec9, and prec11) were retained for constructing the species distribution model (**Table 1**).

**Table 1:** Screened environmental factors and their contribution.

Code	Description	Percent contribution (%)
bio4	Temperature seasonality (standard deviation ×100)	28.2
prec5	May precipitation	24.2
bio2	Mean diurnal range	20.4
bio7	Temperature annual range	10.2
prec11	November precipitation	3.8
prec4	April precipitation	3.7
prec3	March precipitation	2.9
prec6	June precipitation	2.6
elev	elevation	2.1
prec9	September precipitation	1.1
slope	slope	0.4
bio14	Precipitation of driest month	0.3

### 2.3 Maxent modeling and optimization

Distribution records of *L. japonicum* and the filtered environmental variables were imported into the MaxEnt model to simulate its suitability zones. In this study, 75% of the location data was used for training, and the remaining 25% for testing. The bootstrap procedure was repeated 10 times (Phillips et al., 2017). The number of iterations was set to 10,000 to enhance model accuracy (Lu et al., 2024).

The ROC curve is widely used to evaluate species’ potential distribution prediction models, with its area under the curve (AUC) ranging from 0 to 1. A higher AUC value indicates a stronger correlation between the model’s predicted species distribution and environmental factors, signifying better predictive performance. The accuracy characterized by AUC is categorized as follows: 0.50-0.60 (failing), 0.60-0.70 (poor), 0.70-0.80 (fair), 0.80-0.90 (good), and 0.90-1.00 (excellent) (Hanley and McNeil, 1982). In addition to AUC, the True Skill Statistic (TSS) was also employed to evaluate model performance, as it accounts for both sensitivity and specificity, offering a more balanced assessment of predictive accuracy (Allouche et al., 2006). Generally, TSS values below 0.40 indicate poor model performance, values between 0.40 and 0.75 are considered good, and values exceeding 0.75 are regarded as excellent (Dai et al., 2018).

MaxEnt models typically use default parameters, but studies have shown that unadjusted models may perform poorly (Morales et al., 2017; Ma and Sun, 2018). Therefore, optimizing model parameters to reduce overfitting is essential (Fan and Luo, 2024), as optimized MaxEnt models significantly improve prediction accuracy, yielding more reliable species distribution maps (Yüksel and Ambarli, 2024). To achieve this, we used the Kuenm package (https://github.com/marlonecobos/kuenm) in R version 4.2.3 (https://www.r-project.org/) to optimize the regularization multipliers (RM) and feature class parameters (FC) for building the optimal model (Cobos et al., 2019). A total of 1240 candidate models were evaluated, incorporating all combinations of 40 regularization multiplier settings (ranging from 0.1 to 4, in intervals of 0.1) and 31 feature class combinations. The model selection process prioritized statistical significance (partial ROC), predictive ability (low omission rates), and complexity (AICc values), in that order. Initially, candidate models were filtered to retain those that were statistically significant. The models were then further reduced by applying the omission rate criterion (targeting < 5% where possible). Finally, the model with the lowest ΔAICc value was chosen. Models with ΔAICc < 2 were regarded as reliable, with the model exhibiting the smallest ΔAICc (ΔAICc = 0) considered the final model.

### 2.4 Regional analysis of suitability

The results of the MaxEnt model were imported into ArcGIS for format conversion and reclassification. The suitability areas of *L. japonicum* were classified into four categories using the natural breakpoint method: unsuitable areas (p < 0.09874), low suitability areas (0.09874 ≤ p < 0.292655), medium suitability areas (0.292655 ≤ p < 0.490096), and high suitability areas (p ≥ 0.902606) (Nan et al., 2024). The area of suitability zones for *L. japonicum* was also calculated and analyzed. To further investigate changes in the high suitability areas of *L. japonicum*, we used the SDM toolbox (Brown et al., 2017) in ArcGIS to visualize changes in area and the migration of centroids in high suitability zones under current and future climate scenarios.

## 3 Results

### 3.1 Optimal modeling and accuracy assessment

**Figure 3:**
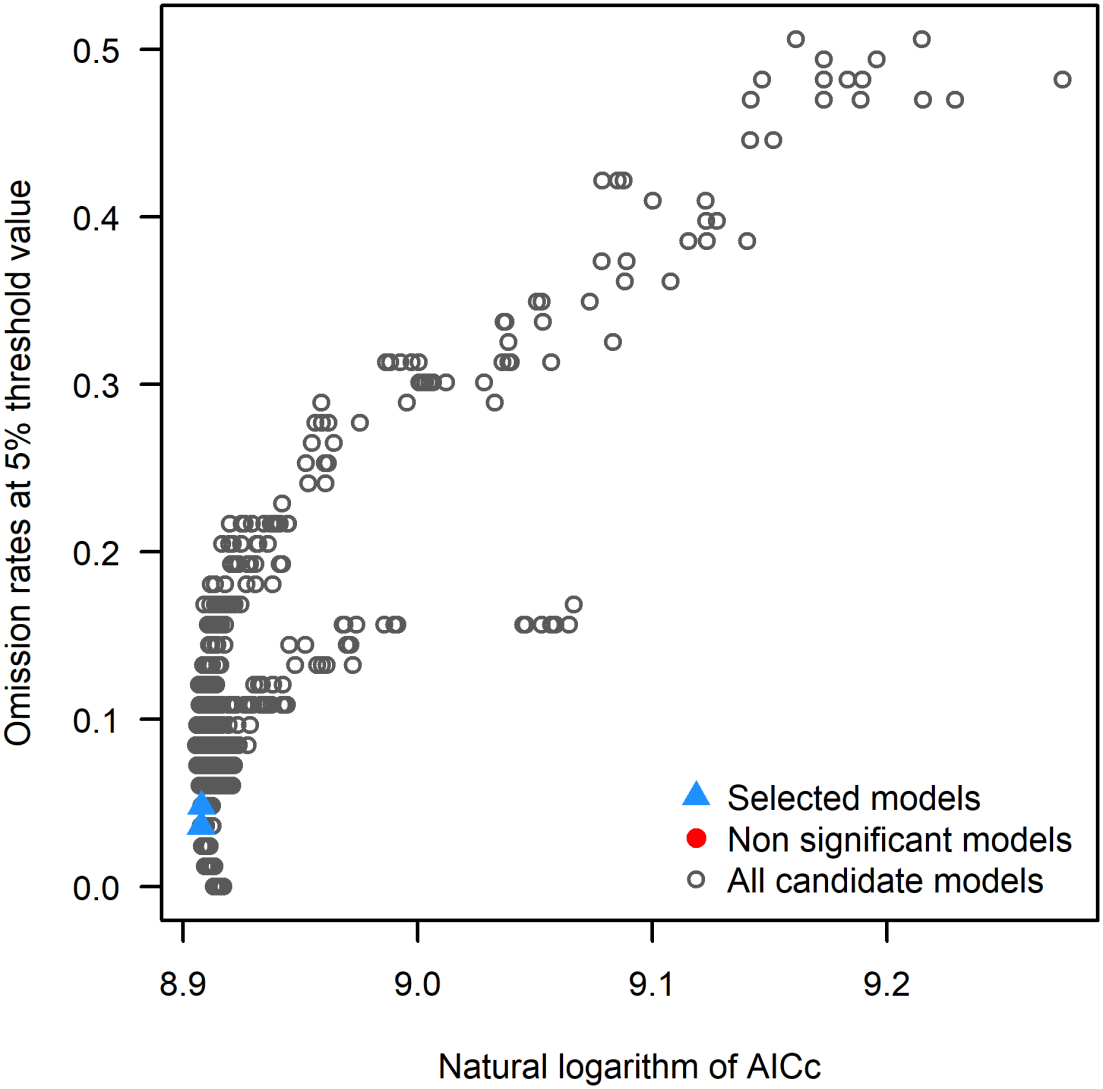
Results of MaxEnt model parameter optimization.

**Figure 4:**
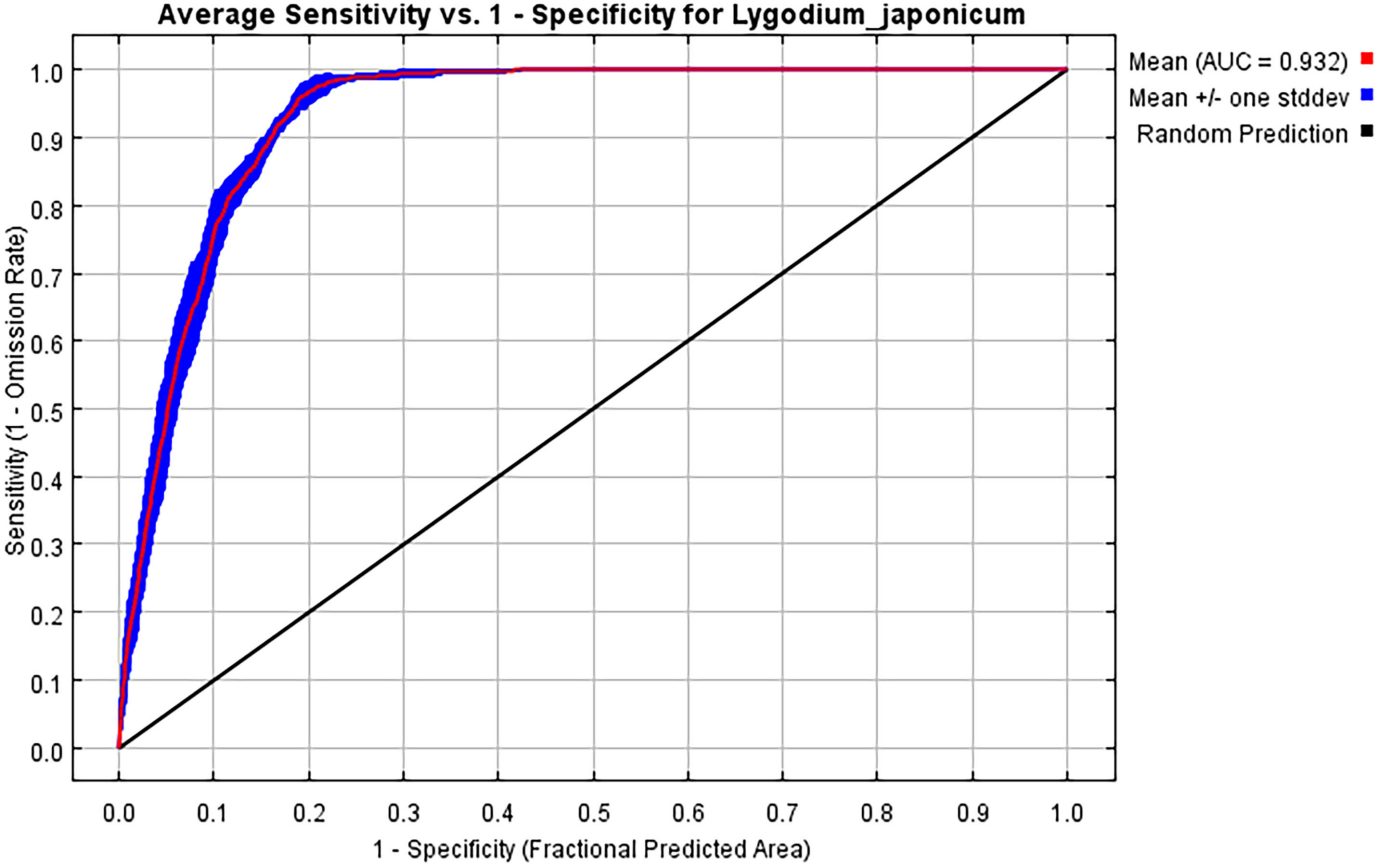
ROC curve response under the MaxEnt model.

Based on 337 distribution records of *L. japonicum* and 12 environmental factors, the potential distribution area of *L. japonicum* was simulated using the MaxEnt model. When the model was run with default parameters, ΔAICc was 70.79. However, after parameter tuning to FC = lp and RM = 3.6, ΔAICc was reduced to 0, indicating that this was the optimal model (**Figure 3**). This optimization enhanced prediction accuracy while mitigating overfitting. Optimizing the model prior to predicting the suitable habitat of *L. japonicum* under different climate scenarios resulted in greater accuracy compared to using the default parameters. With the optimized settings (FC = lp, RM = 3.6), the model achieved an AUC value of 0.932 (**Figure 4**) and an average TSS value of 0.794, both of which reflect excellent model performance and strong predictive reliability.

### 3.2 Current and future habitat suitability for *L. japonicum* in China

Based on the optimized MaxEnt model, the potential distribution of *L. japonicum* in China was projected under both current and future climate scenarios (2041-2060 and 2061-2080). The extent of suitable habitat area across different periods is presented in **Table 2**. Under current climatic conditions, the potential habitat for *L. japonicum* in China spans approximately 216.31 × 10^4^ km², accounting for about 22.53% of the total land area. High suitability areas cover 64.37 × 10^4^ km² (6.71% of the country), primarily concentrated in Hunan, Guangxi, eastern Guizhou, southern Chongqing, the border of Sichuan and Guizhou, and parts of Hubei, Anhui, Jiangxi, Guangdong, Yunnan, Zhejiang, Hainan, and Taiwan. The medium suitability zones extend outward from the high suitability areas, covering 74.03 × 10^4^ km² (7.71%) and including parts of Sichuan, Chongqing, Hubei, Anhui, Jiangsu, Zhejiang, Hainan, and several other provinces. The low suitability areas, covering 77.9 × 10^4^ km² (8.12%), are primarily located at the periphery of the medium suitability zone, including southern Tibet, southern Yunnan, eastern Sichuan, and parts of other provinces. These findings underscore the broad ecological adaptability of *L. japonicum* across China, with higher suitability predominantly observed in the southern and eastern regions. The current distribution of potential habitats is depicted in **Figure 1**.

**Table 2:** Area of suitable habitats for different time periods (×10^4^ km²).

Time	Not Suitable	Low Suitability	Moderate Suitability	High Suitability
Current	743.69	77.9	74.03	64.37
SSP126(2041-2060)	751.43	88.13	84.44	36
SSP126(2061-2080)	748.18	78.72	76.36	56.74
SSP245(2041-2060)	741.69	87.16	87.91	43.24
SSP245(2061-2080)	743.22	89.73	87.25	39.79
SSP585(2041-2060)	744.95	82.25	82.13	50.01
SSP585(2061-2080)	747.28	82.18	78.44	52.1

**Figure 5:**
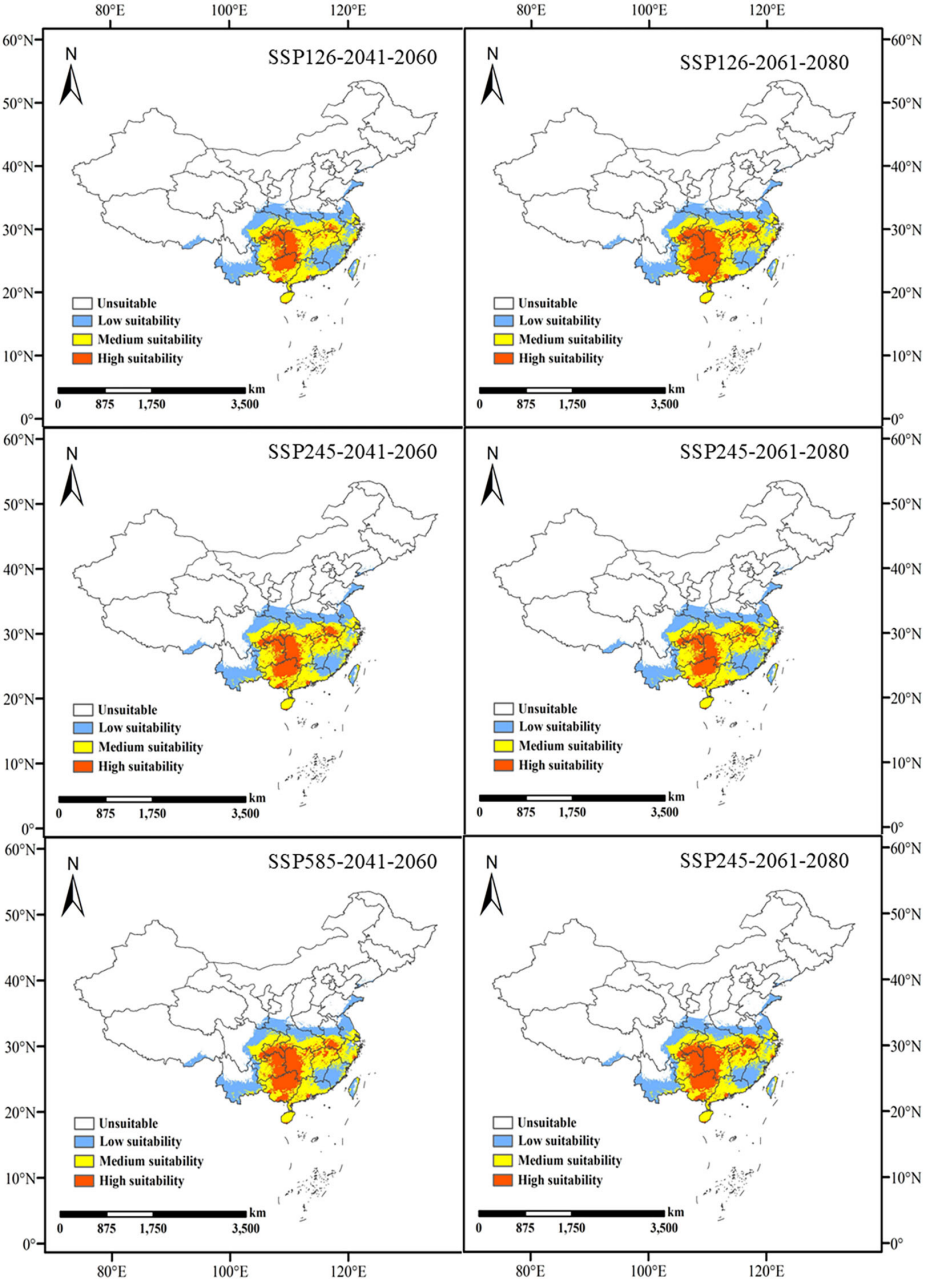
Predicted future habitat distribution of *L. japonicum*.

Under the SSP126 scenario, the suitable habitat area is projected to decrease from the current 216.31 × 10^4^ km² to 208.57 × 10^4^ km², decreasing further to 211.82 × 10^4^ km² in 2061-2080, indicating an overall declining trend. This suggests that *L. japonicum* will continue to face climate pressures. Under the SSP245 scenario, the suitable habitat area initially increases slightly to 218.31 × 10^4^ km², then slightly decreases to 216.77 × 10^4^ km² in 2061-2080. This indicates that in more moderate emission scenarios, suitable habitats may expand, but climate change will still impact them. Under the SSP585 scenario, the suitable habitat area is projected to decrease to 214.39 × 10^4^ km² and further decline to 212.72 × 10^4^ km², showing a clear downward trend. This highlights the negative impact of extreme emission scenarios on *L. japonicum* habitats. The potential future habitat distribution of *L. japonicum* is shown in **Figure 5**.

### 3.3 Comparison of high suitability areas in current and future scenarios

**Figure 6:**
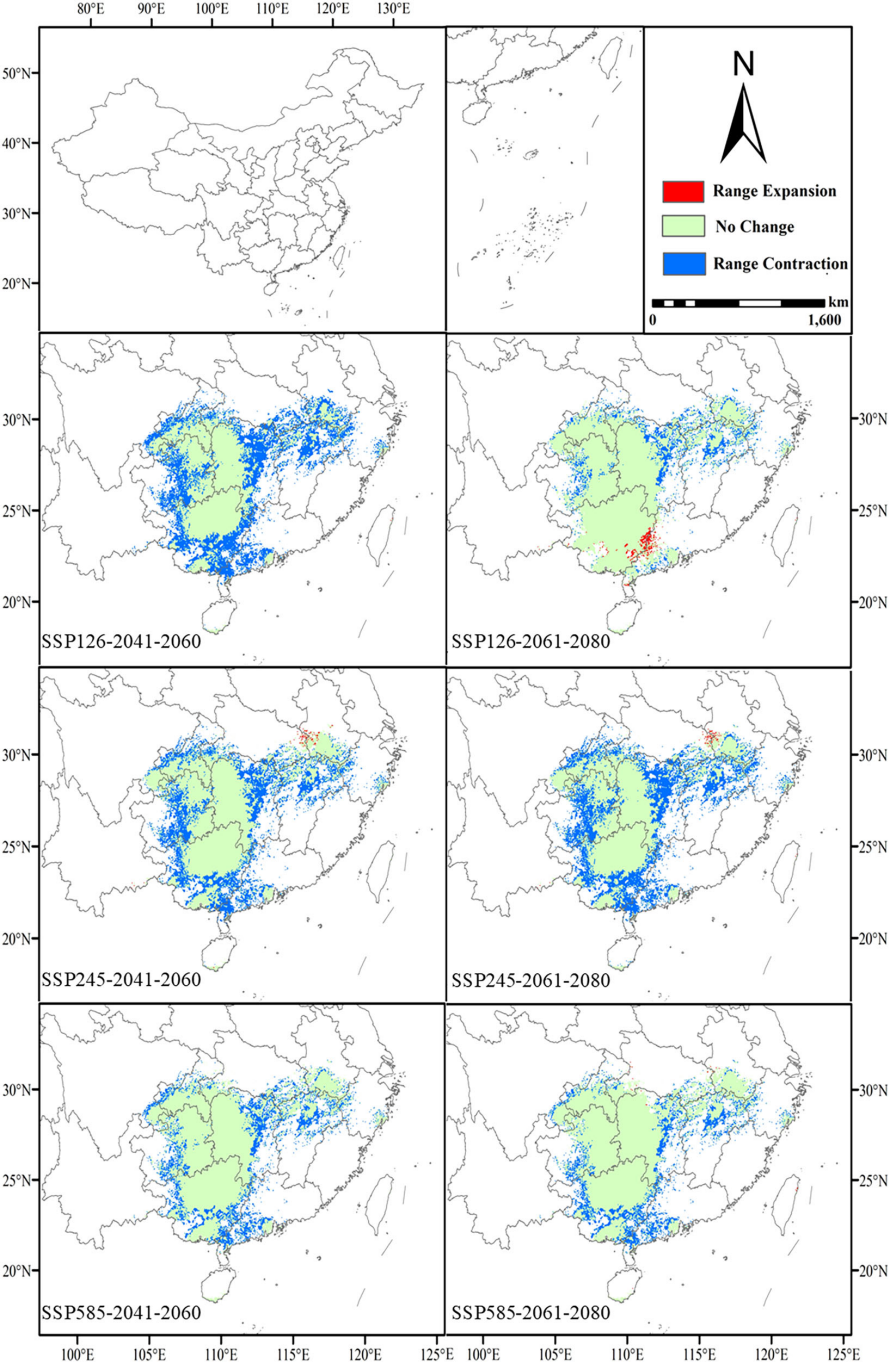
Future changes in the geographic and spatial patterns of high suitability areas compared to the present.

The high suitability zones in all future scenarios generally show a narrowing trend compared to the present, with the most pronounced changes observed in the SSP126 and SSP585 scenarios (**Figure 6**). Under the SSP126 scenario, the high suitability area decreases from the current 64.37 × 10^4^ km² to 36 × 10^4^ km² (a decrease of 44.1%) and further shrinks to 56.74 × 10^4^ km² (a decrease of 11.9%) in 2061-2080. Although there is an overall reduction, high suitability areas in Yunnan, Taiwan, Guangxi, Guangdong, and the border regions between Guangxi and Guangdong increase during 2061-2080. This suggests that, despite the impacts of climate change, some regions may still offer new suitable habitats for *L. japonicum*. Under the SSP245 scenario, the high suitability area first decreases to 43.24 × 10^4^ km² (a decrease of 32.8%) and then further declines to 39.79 × 10^4^ km² (a decrease of 38.2%). Compared to the current situation, areas of increased high suitability are primarily found at the border between Hubei and Anhui, suggesting that localized regions may provide new suitable habitats for *L. japonicum* under certain climate change conditions. Under the SSP585 scenario, the high suitability area initially decreases to 50.01 × 10^4^ km² (a decrease of 22.3%) and then further reduces to 52.10 × 10^4^ km² (a decrease of 19.1%). The newly identified high suitability areas during 2061-2080 are mainly scattered across Hainan, Taiwan, Hubei, Hunan, Sichuan, and Anhui, reflecting regional variations under extreme climate scenarios.

### 3.4 Centroid migration in high suitability area

**Figure 7:**
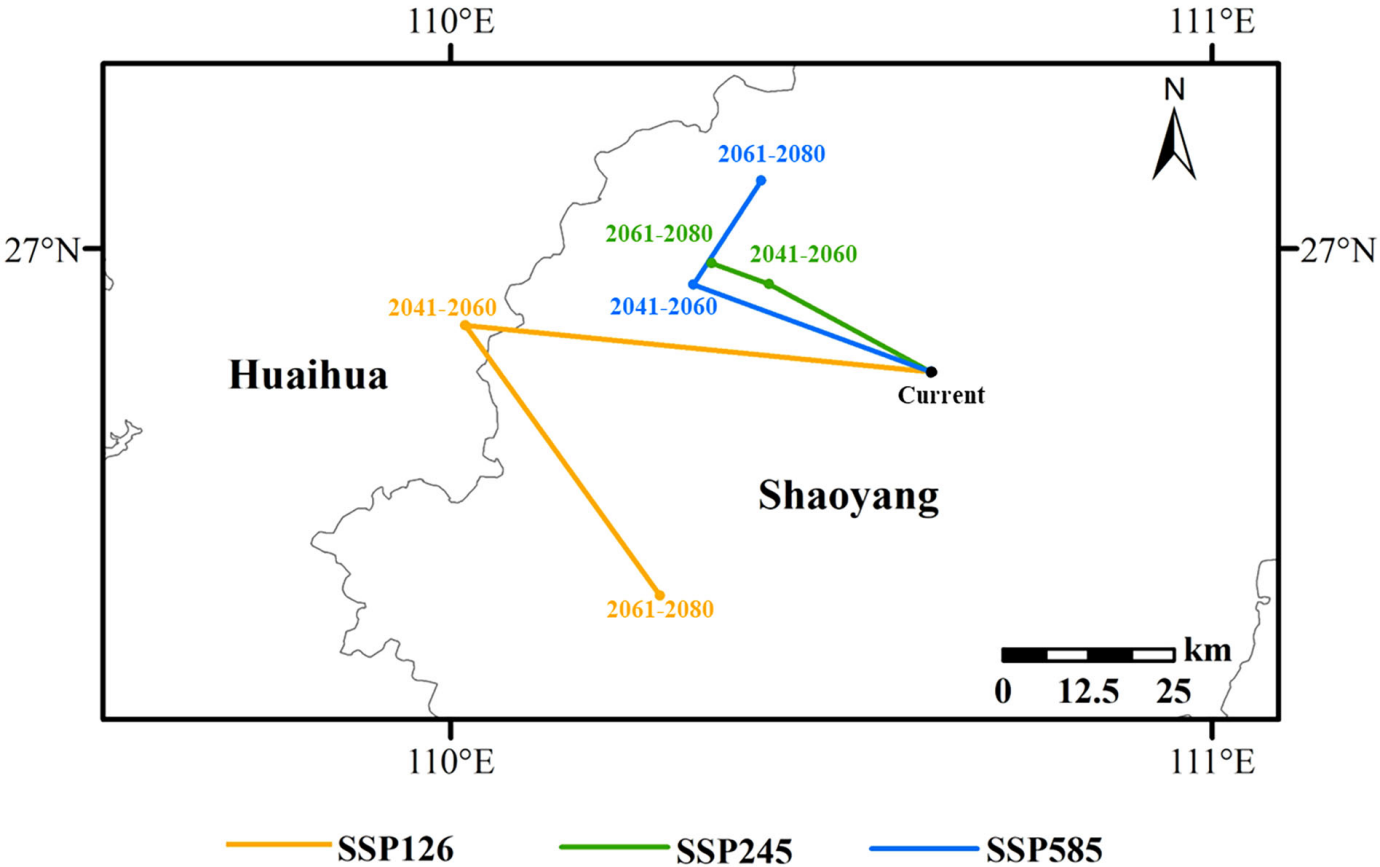
Migration pathways of *L. japonicum* distribution centroids.

The centroids of the high suitability areas under the current and future scenarios are all located in Hunan Province. The centroid of the current high suitability area of *L. japonicum* is marked as a black dot, located in Wugang City, Shaoyang, Hunan Province (110°37’51”E, 26°50’17”N). From the present to 2041-2060, the centroids under the three scenarios (SSP126, SSP245, and SSP585) shift northwestward by 59.46 km, 24.40 km, and 32.81 km, respectively. The displaced centroids are located in Huaihua City, Huitong County (110°1’8”E, 26°53’58”N), Shaoyang City, Suining County (110°25’4”E, 26°57’13”N), and Shaoyang City, Suining County (110°19’8”E, 26°57’10”N). From 2041-2060 to 2061-2080, the centroid under the SSP126 scenario will migrate southeastward by 47.49 km to Suining County, Shaoyang City (110°16’31”E, 26°32’41”N); under the SSP245 scenario, the centroid will shift northwestward by 7.9 km to Suining County, Shaoyang City (110°20’34”E, 26°58’52”N); under the SSP585 scenario, the centroid will move northeastward by 17.78 km to Dongkou County, Shaoyang City (110°24’28”E, 27°5’22”N) (**Figure 7**).

### 3.5 Main environmental factors affecting the distribution of *L. japonicum*

**Figure 8:**
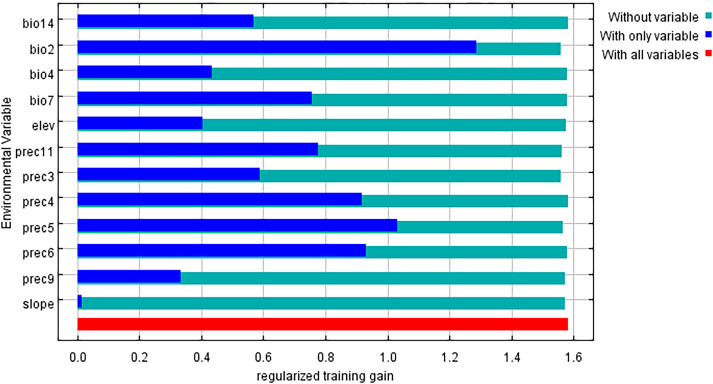
Jackknife test of regularized training gain for environmental variables.

The key environmental variables influencing the distribution of *L. japonicum* were primarily evaluated through the relative contributions of environmental variables to the Maxent model and the results of the jackknife test. According to the results (**Table 1**), bio4 (28.2%), prec5 (24.2%), and bio2 (20.4%) were the main factors in constructing the model, with a cumulative contribution of 72.8%. The less influential environmental variables included bio7 (10.2%), prec11 (3.8%), prec4 (3.7%), prec3 (2.9%), prec6 (2.6%), elevation (2.1%), prec9 (1.1%), slope (0.4%), and bio14 (0.3%), collectively contributing 27.1%. The results of assessing the gain effect of the environmental variables through the jackknife method are shown in **Figure 8**. As shown in the figure, the most prominent variables are bio2 and prec5, indicating that these two factors have the greatest influence on the distribution of suitable areas for *L. japonicum*. In summary, we conclude that bio4, bio2, and prec5 are the key factors affecting the distribution of suitable areas for *L. japonicum*.

**Figure 9:**
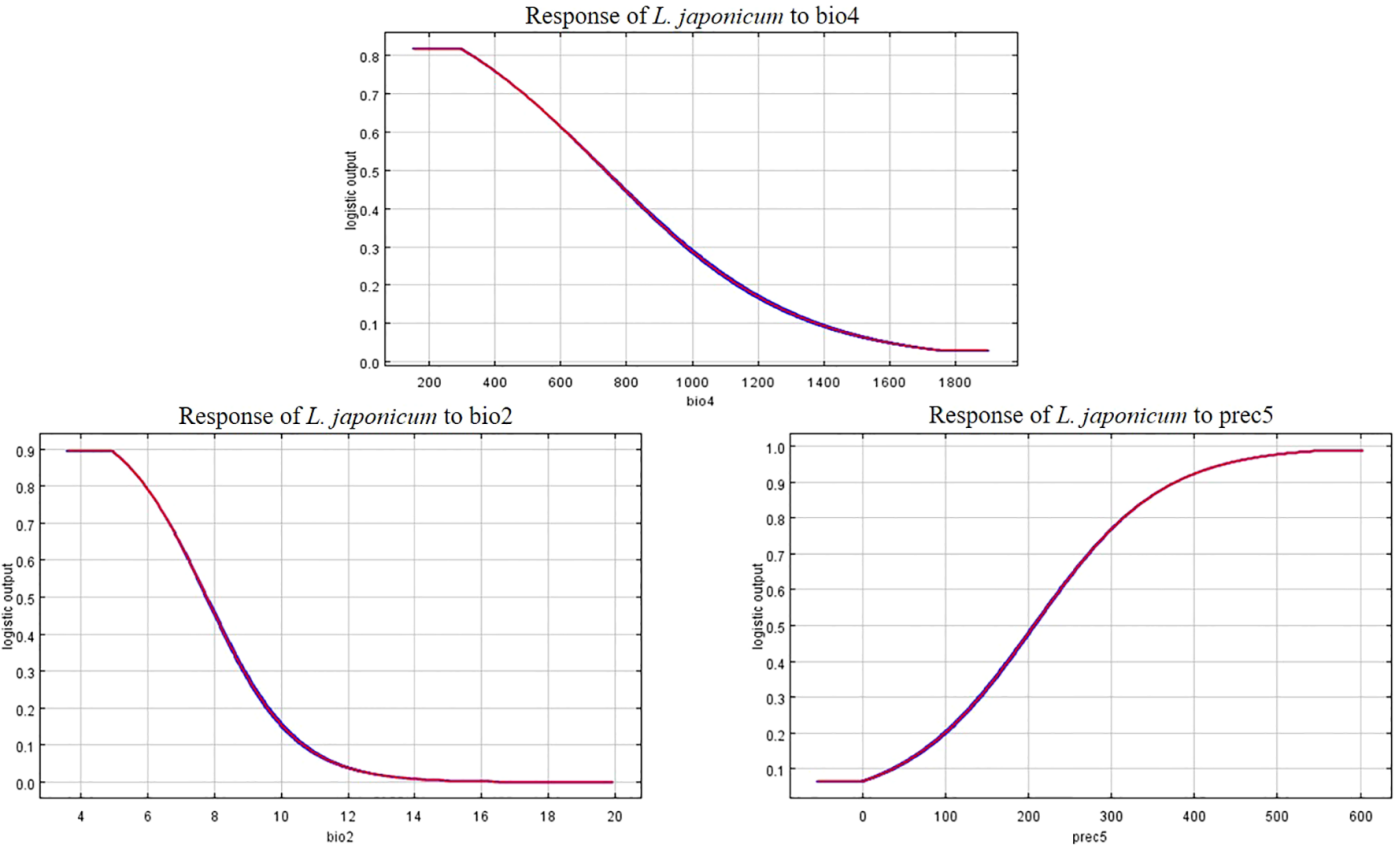
Response curves of key environmental factors in the species distribution model for *L. japonicum*.

The response curves of the three key environmental factors significantly influencing the distribution of *L. japonicum* clearly illustrate the relationship between the species’ suitability and these dominant environmental factors (**Figure 9**). A suitability probability greater than 0.5 is typically considered the most favorable range for the species’ growth. For bio2 (mean diurnal range), the species maintains a high suitability probability (0.9) when the value is between 3.5 and 5. As the value increases beyond 5, the probability gradually decreases, reaching 0.5 at 7.7. For prec5 (May precipitation), the suitability probability exceeds 0.5 when precipitation exceeds 207 mm, extending up to approximately 600 mm. Finally, for bio4 (temperature seasonality), the species shows high suitability (0.82) when bio4 ranges from 152 to 300, with suitability dropping to 0.5 when bio4 exceeds 750.

## 4 Discussion

### 4.1 Model assessment

As climate change intensifies, predicting species distributions has become increasingly crucial for biodiversity conservation and ecosystem management (Jiang et al., 2016). Climate models offer valuable insights into how changes in environmental conditions may impact species’ habitats, thus enabling the development of more informed conservation strategies. For *L. japonicum*, understanding its future distribution is particularly vital for its growing demand in traditional Chinese medicine and its limited resources. These pressures underscore the importance of protecting its natural habitat, as shifts in its suitable distribution range could complicate sustainable harvesting and conservation efforts. Maxent modeling is a widely used tool for predicting plant distributions, particularly because of its ability to handle presence-only data and its relatively high predictive accuracy (Phillips and Dudík, 2008). In this study, Maxent was applied to forecast the potential growing areas of *L. japonicum* in China. The model was optimized with ΔAICc = 0, achieving an AUC of 0.932 and a TSS of 0.794, both indicating excellent predictive performance and robustness, thereby validating the model’s suitability for climate-based habitat forecasting.

### 4.2 Environmental factors influencing the distribution of *L. japonicum*

In this study, we examined three key environmental factors—mean daily temperature variation (bio2), May precipitation (prec5), and temperature seasonality (bio4)—that significantly influence the distribution of *L. japonicum*. As an herbaceous plant, *L. japonicum* thrives in warm, humid climates, where both rainfall and temperature play crucial roles in its growth. From an ecological perspective, bio2 reflects the impact of temperature fluctuations on plant growth. Within a suitable range of temperature variation (3.5 to 7 °C), *L. japonicum* is better able to adapt to environmental changes. Specifically, when the diurnal temperature variation is between 3.5 and 5°C, the temperature fluctuations are moderate, supporting higher growth suitability. However, when temperature differences exceed 7°C, the species’ growth suitability declines. This may be attributed to the negative effects of larger diurnal temperature fluctuations on plant physiological functions. For instance, higher diurnal temperature differences in some tree species exacerbate drought stress, reduce stomatal conductance, and ultimately limit growth (Zhang et al., 2023). For prec5 (May precipitation), the suitability probability exceeds 0.5 when precipitation is greater than 207 mm, remaining high up to approximately 600 mm. This indicates that *L. japonicum* requires adequate moisture early in the growing season for germination and early growth, with precipitation patterns playing a crucial role in determining its distribution, especially in the context of climate change (Yan et al., 2015). Areas receiving less than 207 mm of rainfall in May may experience moisture stress during critical growth phases, limiting the species’ ability to thrive. Regarding bio4 (temperature seasonality), our results show that *L. japonicum* exhibits high growth suitability in areas with moderate temperature fluctuations (152-300). This suggests that the species is well-adapted to regions with stable seasonal temperatures. In contrast, regions with extreme temperature seasonality (bio4 > 750) have reduced suitability, likely due to the adverse effects of sharp seasonal temperature differences on the plant’s growth and distribution.

### 4.3 Spatial patterns of suitable areas

Under current climatic conditions, the potential habitat for *L. japonicum* in China spans approximately 216.31×10^4^ km², accounting for roughly 22.53% of the country’s total land area. These suitable areas are primarily found in several provinces, including Hunan, Guangxi, southern Guizhou, southern Chongqing, Sichuan, Hubei, Anhui, Jiangxi, Guangdong, Yunnan, Zhejiang, Hainan, and Taiwan. High suitability areas are concentrated in regions such as Hunan, Guangxi, southern Guizhou, southern Chongqing, the Sichuan-Guizhou border, and parts of Hubei, Anhui, Jiangxi, Guangdong, Yunnan, Zhejiang, Hainan, and Taiwan, covering 6.71% of the country’s total land area. These distribution results suggest that *L. japonicum* is highly suitable for cultivation in southern and eastern China, with suitable habitats widely dispersed across several provinces, particularly in the southern and southeastern coastal regions. Projections under future climate scenarios indicate that, while the area of suitable habitat in China may decrease in some regions and increase in others, the overall change in area is not dramatic, and the general distribution pattern remains relatively stable.

Under future climate scenarios, the high suitability areas of *L. japonicum* generally show a contraction. However, localized increases in high suitability areas are observed in certain regions. During 2061-2080, under the SSP126 scenario, new high suitability areas emerge along the border between Guangxi and Guangdong. In the SSP245 scenario, new high suitability areas are observed, particularly along the border between Hubei and Anhui. The SSP585 scenario shows smaller, more scattered high suitability areas in regions such as Hainan, Taiwan, Hubei, Hunan, Sichuan, and Anhui. This indicates that under moderate climate change conditions, certain areas could still provide suitable habitats.

### 4.4 Future migration of the center of mass in the high suitability zone

Most studies suggest that under future climate change, especially global warming, plant species tend to shift their distributions toward higher latitudes (Oke et al., 2023; Rana et al., 2022). The results of this study align with this general trend, as the centroid of *L. japonicum’s* high suitability areas predominantly shifts northward under most climate scenarios (SSP126, SSP245, and SSP585). However, in the SSP126 scenario for the period 2061-2080, the centroid shifts southeast instead. This southward shift contrasts with the typical northward movement expected under climate change and has been observed in other plant studies as well (Chen et al., 2024; Jiang et al., 2023). The southward migration in this case can be attributed to the emergence of new high suitability areas in southern regions such as Guangxi, Guangdong, and Hainan. Under moderate climate scenarios, some regions may become more favorable for *L. japonicum* due to changes in temperature and precipitation, leading to a southward shift in its centroid. This challenges the common expectation of northward migration and highlights the regional variability in species’ responses to climate change. The shift indicates that climate change may drive both southern expansion and northern contraction, depending on emission scenarios and ecological conditions, emphasizing the need for further research.

## 5 Conservation strategies and implications

Ensuring the sustainability of *Lygodium japonicum* populations, both in the wild and in cultivation, requires conservation strategies that consider the impacts of climate change and predicted spatial dynamics. Key strategies include habitat restoration in areas projected to remain suitable and establishing protected areas to prevent overcollection. Currently, conservation efforts should prioritize high suitability regions such as Hunan, Guangxi, southern Guizhou, and southern Chongqing, focusing on habitat protection, early monitoring, and sustainable harvesting regulation to detect and respond to population declines promptly. Promoting cultivation in climatically stable areas will alleviate pressure on wild populations and meet market demand. Additionally, emerging suitable habitats under future climate scenarios, such as the Guangxi–Guangdong border under SSP126, the Hubei–Anhui border under SSP245, and parts of Hainan and Taiwan under SSP585, require targeted habitat restoration and monitoring to facilitate species expansion and adaptation. Further research on *L. japonicum’s* response to environmental changes will provide valuable insights to refine these conservation and cultivation strategies, enhancing the species’ resilience and sustainable use.

## 6 Research limitations

Although this study provides important insights into the potential distribution of *L. japonicum*, some limitations should be considered. First, the environmental variables used in the model did not fully account for anthropogenic factors, such as land-use change, pollution, and habitat fragmentation, which may also influence the species’ distribution. Incorporating these factors into future models could improve prediction accuracy and offer a more comprehensive understanding of the species’ ecological requirements. Additionally, the model assumes that the ecological niche of *L. japonicum* will remain relatively stable over time. However, evolutionary and adaptive responses to changing climatic conditions may alter its distribution. Longitudinal field and experimental studies on the species’ adaptations to climatic stressors will be valuable in refining our predictions.

## 7 Conclusions

This study provides valuable insights into the future distribution of *L. japonicum* under climate change, emphasizing its conservation needs. Using the Maxent model, we identified key environmental factors, including temperature variation (bio2), May precipitation (prec5), and temperature seasonality (bio4), that influence habitat suitability. Currently, its potential habitat spans 216.31 × 10^4^ km², primarily in southern and eastern China. While the overall distribution remains stable under future climate scenarios, high suitability areas are expected to shrink, though some regions, particularly in the south, may experience localized expansions. The centroid is projected to shift northward, with some southward shifts observed in the SSP126 scenario. These findings underscore the need for proactive conservation strategies, including habitat restoration and cultivation in stable regions, to mitigate the impacts of climate change. Further research on anthropogenic factors will be essential to refine conservation efforts for *L. japonicum*.

## Data Availability

The original contributions presented in the study are included in the article/supplementary material. Further inquiries can be directed to the corresponding authors.
